# Embryonic and adult synaptic proteome perturbations after maternal immune activation: Identification of persistent changes relevant for early intervention

**DOI:** 10.21203/rs.3.rs-3100753/v1

**Published:** 2023-07-03

**Authors:** Anna Y. Yotova, Li-Li Li, Aet O’Leary, Irmgard Tegeder, Andreas Reif, Michael J Courtney, David A. Slattery, Florian Freudenberg

**Affiliations:** 1 Goethe University Frankfurt, University Hospital, Department of Psychiatry, Psychosomatic Medicine and Psychotherapy, Frankfurt, Germany; 2 Goethe University Frankfurt, Faculty of Biological Sciences, Institute of Cell Biology and Neuroscience, Frankfurt, Germany; 3 Turku Bioscience Centre, University of Turku and Åbo Akademi University, 20520 Turku, Finland; Turku Brain and Mind Center, University of Turku and Åbo Akademi University, 20014, Turku, Finland; 4 Department of Neuropsychopharmacology, Institute of Chemistry, University of Tartu, Tartu, Estonia; 5 Goethe University Frankfurt, Faculty of Medicine, Institute of Clinical Pharmacology, Frankfurt, Germany

## Abstract

Maternal infections during pregnancy pose an increased risk for neurodevelopmental psychiatric disorders (NPDs) in the offspring. Here, we examined age- and sex-dependent dynamic changes of the hippocampal synaptic proteome after maternal immune activation (MIA) in embryonic and adult mice. Adult male and female MIA offspring exhibited social deficits and sex-specific depression-like behaviours, among others, validating the model. Furthermore, we observed dose-, age-, and sex-dependent synaptic proteome differences. Analysis of the embryonic synaptic proteome implicates sphingolipid and ketoacid metabolism pathway disruptions during neurodevelopment for NPD-pertinent sequelae. In the embryonic hippocampus, prenatal immune activation also led to changes in neuronal guidance, glycosphingolipid metabolism important for signalling and myelination, and post-translational modification of proteins that regulate intercellular interaction and developmental timing. In adulthood, the observed changes in synaptoneurosomes revealed a dynamic shift toward transmembrane trafficking, intracellular signalling cascades, and hormone-mediated metabolism. Importantly, 68 of the proteins with differential abundance in the embryonic brains of MIA offspring were also altered in adulthood, 75% of which retained their directionality. These proteins are involved in synaptic organisation, neurotransmitter receptor regulation, and the vesicle cycle. A cluster of persistently upregulated proteins, including AKT3, PAK1/3, PPP3CA, formed a functional network enriched in the embryonic brain that is involved in cellular responses to environmental stimuli. To infer a link between the overlapping protein alterations and cognitive and psychiatric traits, we probed human phenome-wise association study data for cognitive and psychiatric phenotypes and all, but PORCN were significantly associated with the investigated domains. Our data provide insights into the dynamic effects of an early prenatal immune activation on developing and mature hippocampi and highlights targets for early intervention in individuals exposed to such immune challenges.

## INTRODUCTION

Recent insights from large-scale systematic studies identify psychiatric disorders as one of the leading causes of the global burden of disease, and their prevalence as not only age- but also sex- and gender-specific^1,2^. Since growing evidence about the pleiotropic nature of mental illness and the major role of disrupted brain development therein emerged^3–7^, neuropsychiatric developmental disorders (NPD) have increasingly occupied a more central role in psychiatric research. A viral infection during pregnancy is an established risk factor for NPDs, including schizophrenia (SCZ), autism spectrum disorder (ASD), and attention-deficit/hyperactivity disorder (ADHD), in the offspring^8–11^. Neuroinflammation, especially during gestation, has gained more prominence in recent years as developmental and social deficits have been observed in infants after a maternal infection during pregnancy^12,13^. Animal and cell models of maternal immune activation (MIA), mimicking infections during pregnancy, have been developed and validated in the past two decades, elucidating the risk of NPD-relevant behavioural, functional, morphological, and molecular deficits in affected offspring^14–16^. Among the most reproducible manifestations of early-to-mid pregnancy viral-like MIA are social deficits, whereas impairments in sensorimotor gating, hippocampus (HPC)-dependent memory and novelty responses are more variable^17,18^

Hippocampal dysfunction has been implicated in multiple behavioural aberrations observed in mental disorders, e.g. ASD and SCZ, including social and spatial memory deficits, novelty response and cognitive impairments^19–22^. Neuroinflammation, especially during gestation, has deleterious effects on HPC morphology, neurogenesis, and function^23–26^, particularly at the synaptic level^27–29^. Such developmental disruptions play a central role in NPD susceptibility as shown in both human^30–32^ and rodent^27,33–35^ studies^14,29^. However, unbiased investigations of how MIA affects the brain on a synaptic level are still missing.

Gestational transcriptional changes following MIA in rodents have been previously reported in whole embryos or embryonic brains, though without examining^36–38^ or identifiying^39^ sex differences. These studies revealed general disruptions in neuroprotective function, neuronal development, metabolism and migration, in addition to the expected neuroinflammatory processes, which are especially pronounced with regard to microglia function^40^. Others have primarily utilised RNA sequencing to probe transcriptomic changes in adult male frontal cortex^16,41,42^, striatum^41^, and amygdala^16,43^ following MIA at different gestational time-points. Neuronal signalling pathway alterations emerged as a common denominator, in addition to changes in myelin and oxidative phosphorylation, among other more subtle region-specific effects. One study combining male and female adolescent mice found increased changes in HPC transcripts after early gestational MIA, disrupting signalling pathways involved in apoptosis, embryo- and synaptogenesis^44^.

Recent studies implementing single-cell RNA sequencing and chromatin accessibility assays largely focused on the role of microglia and other neuroimmune cells in MIA-linked outcomes, comparing the neuroglial profiles of new-born and adult brains, as well as embryonic cell cultures^45,46^. Another study in male mice concentrating on HPC glutamatergic neurons, provided *in vitro* and *ex vivo* evidence that embryonic HPC synaptogenesis plays a role in MIA-relevant aberrations^47^. Moreover, after mid-gestational MIA, late foetal cortical transcriptome analyses have revealed sex-dependent post-transcriptional aberrations^48^. However, these studies did not investigated if, and which, perinatal transcriptome changes persevere into adulthood, which is the crucial question when it comes to the underlying mechanisms of persistent NPDs and, thus, potential treatment targets.

Most recent investigations have utilised transcriptomic methods to explore gene expression changes after a prenatal immunostimulation^49^, but sex-specific findings have remained inconsistent and sporadic. However, given the prominence of sex differences in mental disorders, this issue is of high relevance. So far, available studies have examined either hypothesis-derived genes and pathways^50–55^ or cell- or region-specific RNA transcripts as described above. Importantly, RNA and protein levels often do not correlate^56^ and the brain, specifically, exhibits a large number of distinct protein expression profiles not detectable at the RNA level, including for neurotransmission-relevant vesicle transport genes^57^. To the best of our knowledge, MIA-evoked changes at the functional proteomic level have not been assessed at separate ages in a sex-specific manner^58^.

To address these knowledge gaps, we investigated and compared the proteomic changes in embryonic (gestational day E18) and adult hippocampal synaptoneurosomes of MIA mouse offspring of both sexes, using mass spectrometry. MIA was induced in wild-type pregnant dams (E9) using the viral mimetic polyinosinic:polycytidylic acid [poly(I:C)] injection at two different doses during early gestation to mimic an immune challenge in the late first trimester in humans^59,60^. Moreover, the intervention encompasses a developmental window, during which increased forebrain neurogenesis, initial neuronal migration, and early immune cell colonisation of the brain occur^10,14,46,61^. The model was validated via NPD-relevant behavioural endophenotypes in adult male and female offspring, which then allowed direct comparison of MIA-induced hippocampal synaptic proteome alterations in a sex- and age-dependent manner. Importantly, we aimed to identify persistent proteome changes that could be relevant targeted-intervention strategies for individuals upon early diagnosis or at high risk after MIA.

## MATERIALS AND METHODS

### ANIMALS AND MATERNAL IMMUNE ACTIVATION (MIA)

#### Animals.

All experiments were performed with wild-type *C57BL/6JRj* mice, obtained from Janvier Labs (Le Genest-Saint-Isle, France) or their in-house bred F1 offspring. Experimental mating, MIA, and behavioural testing were performed in adult mice (see Supplementary Methods for more information). All experiments were conducted in accordance with institutional guidelines, current national legislation (*TierSchG, TSchV*), and the EU directive regarding the use of experimental animals for scientific purposes (*86/609/EEC*). Ethical approval was obtained from the local authorities (Regierungspräsidium Darmstadt, animal licence *AZ: FK/1101*).

#### Mating, Pregnancy Determination, and MIA.

Naïve *C57Bl/6JRj* females aged 12±2 weeks were mated overnight with males in a 2:1 female-to-male ratio. Serendipitous discovery of a viscous mucous in the vaginal canal of pregnant dams on E7 was indicative of oestrus cycle cessation, and eliminated false negatives and decreased false positives five-fold (Supplementary Figure S1A-D). These indicators, taken together with maternal weight on E9^62^, were used for pregnancy determination, after which MIA induction commenced.

**Figure 1:**
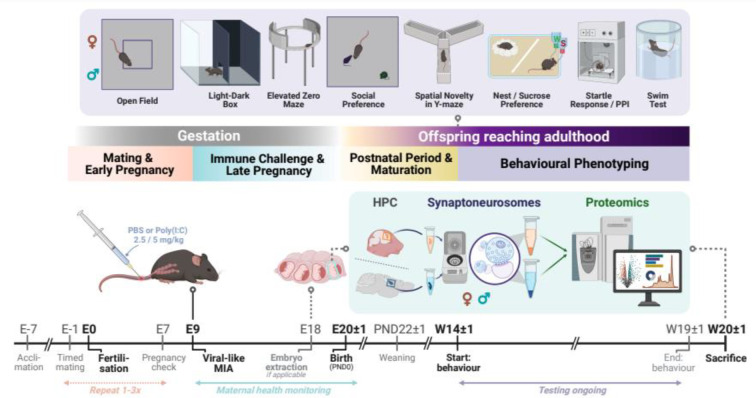
Timeline and summary of experimental design. Maternal immune activation (MIA) was induced by an intravenous administration of 2.5 or 5 mg/kg of the viral-like immunostimulant poly(I:C) in *C57BL/6JRj* mice during early pregnancy (E9). An equivalent vehicle injection of 5 ml/kg PBS was used for controls and all dams’ health was closely monitored. In four separate cohorts, male and female offspring (50±4.5% for each group) were either extracted before birth (E18, n=92) for proteomic analysis in hippocampal synaptoneurosomes (SN), or allowed to reach adulthood. The adult offspring (n=82) was tested using a behavioural test battery for psychiatrically relevant endophenotypes. We assessed locomotion, exploration, and anxiety-like behaviour (Open Field, Light-Dark Box, Elevated Zero Maze), social memory and preference, spatial novelty response and memory (Y-maze), acoustic startle reflex and sensorimotor gating (i.e. prepulse inhibition, PPI), and depressive-like behaviours (Nesting, Sucrose Preference, Swim Test). After a recovery period following the end of testing, mouse brains were collected and SN proteomics in the HPC were performed. For more information on experimental procedure and animals, see Supplementary Table S1, Supplementary Figure S1–2, and Supplementary MIA Checklist. *Abbreviations: E*, embryonic day; *HPC*, hippocampus; *MIA*, maternal immune activation; *PBS*, phosphate-buffered saline; *poly(I:C)*, polyinosinic:polycytidylic acid.

MIA was induced on E9 of the putative pregnancy according to Meyer et al., 2005^63^ by administration of the viral mimetic polyinosinic:polycytidylic acid [poly(I:C)], a synthetic RNA immunostimulant that binds to toll-like receptor 3 (TLR3) and induces transient infectious-like states by cytokine pathway activation^64^. Dams were injected with vehicle or equivalent volumes of 2.5 or 5 mg/kg poly(I:C) solutions, prepared from poly(I:C) potassium salt (Sigma-Aldrich, Schnelldorf, Germany). The success of immune activation was further validated in blood plasma collected 1 h after poly(I:C) injection, showing a significant, dose-dependent increase in the relevant pro-inflammatory cytokine interleukin-6 (IL-6)^65^ in an ELISA immunoassay (Supplementary Figure S1E). All resulting offspring (n=4–7 litters/group, 32 surviving litters over three cohorts) were used for either behavioural or embryonic and adult HPC molecular analyses (Supplementary Table 1). More detailed information on mating, immune challenge procedure, and experimental design, according to reporting guidelines^18^, is provided in the Supplementary MIA Checklist and Supplementary Methods (overview and timeline in Figure 1).

### BEHAVIOURAL EXPERIMENTS

Adult offspring (n=82, 40 males, 42 females from 6±1 litters/group; Supplementary Table 1) were phenotyped using a battery comprising nine behavioural tests that were performed in order of increasing aversiveness, starting at 14±1 weeks of age (Supplementary Figure S2A). Locomotion and exploration, anxiety- and depressive-like behaviours, sensorimotor gating, sociability, and memory in different contexts were assessed, adapted from experimental designs published previously^66–68^. See Supplementary Methods for detailed information.

### PROTEOMICS AND BIOINFORMATICS

#### Tissue and Synaptoneurosome (SN) Preparation.

Adult and embryonic (E18) HPC were extracted and stored liquid-free at −80 °C. Additionally, embryonic tail clippings were used to determine the sex by performing a simplex PCR assay (KAPA2G Fast HotStart Genotyping Mix, Thermo Fisher Scientific, Bremen, Germany) as described elsewhere^69^. For SN preparation, a filtration method^70^ was used, similar to previous descriptions^71^. Details on tissue and SN extraction can be found in the Supplementary Methods. In total, four replicates of each control and poly(I:C) offspring were used for proteomic analyses (E18 samples: 14 F, 16 M and 4 replicates per group, n=7–8 embryos/replicate, adult samples: 2 M, 2 F and 4 replicates per group, biological replicates represent individual animals; see also Supplementary Table S1).

#### Protein Digestion and Mass Spectrometry Analysis.

Mass spectrometry analyses were performed at the Turku Proteomics Facility supported by Biocenter Finland. Briefly, 25 or 50 μg of protein for embryos and adults, respectively, were taken for in-solution digestion and subjected to filter-aided sample preparation (FASP)^72^, according to the facilities protocol. Detailed description is provided in the Supplementary Methods.

#### Protein Identification, Differential Abundance, and Clustering Analyses.

Data was processed by Spectronaut (v15.0.2, Biognosys, Schlieren, Switzerland) software and analysis consisted of protein identifications and label-free quantifications of protein abundances. Direct data-independent acquisition (DIA) approach was used to identify proteins and label-free quantifications were performed with the intensity determination and normalization procedure MaxLFQ (see Supplementary Methods for details).

The resulting detected protein lists were then cleaned by removing histones and keratins considered impurities (n=20 in adult, 29 in embryonic data set), and used for all subsequent analyses. All data sets were filtered at biologically relevant thresholds of 20 % absolute change (1.2>fold change, FC >0.8) and multiple testing corrected significance values q<0.05. For visualisation purposes, abundance differences were calculated as *log2*(FC) and significance as negative *log10*(q-value). The heat map and hierarchical clustering of the differentially regulated proteins common to the adult and embryonic MIA datasets was generated using the *pheatmap* R package, where colour scale represents *log2*(FC) in SN protein expression.

#### Enrichment and Network Analyses.

Significantly changed protein sets were compared between groups and visualised using InteractiVenn, an online tool for set analyses, identifying overlapping and unique abundance differences^73^. Functional enrichment was assessed using a web-based toolkit WebGestalt (https://www.webgestalt.org/), selecting *Mus musculus* as organism of interest^74^ (details provided in Supplementary Methods). Enrichment analysis of the full lists of detected proteins in the protein-coding genome was successfully performed for SN preparation validation (Supplementary Table 7). An online platform for synapse research based on published experimental evidence, namely SynGO^75^, was utilised to assess MIA-induced changes in synaptic proteins, specifically. Protein network analysis was performed by STRING (https://string-db.org/; v11.5)^76^. Functional network enrichment was assessed using the respective cleaned background protein list as reference for embryonic and adult datasets.

#### Phenome-wide association analysis (PheWAS).

PheWAS was performed for all genes corresponding to proteins common to the adult and embryonic datasets (n=68), using the genome-wide association study (GWAS) ATLAS database (https://atlas.ctglab.nl; v20191115), based on publicly available data resources.^76^ Associations of the genes coding for the proteins and a total of 399 GWAS-acquired traits across two relevant domains (Psychiatric, n=321; Cognitive, n=78) were probed and significant correlations were extracted (p<0.05). A full list of included domains and traits can be found in Supplementary Table 2.

### STATISTICAL ANALYSES

Proteomic results were analysed for differential abundance using an unpaired *t*-test and multiple-testing correction of the p-values with the Benjamini-Hochberg (B-H) method (q-values). Enrichment of protein abundance differences was calculated with the pertinent online software^74,75^ (Fisher’s exact test for overrepresentation analysis (ORA)/SynGO analyses; B-H corrected). Since adult males and females differ in both baseline behaviour and protein abundance profiles, the consequences of MIA were assessed within each sex individually. Comparisons of MIA-evoked SN proteomic changes for both the adult and embryonic dataset were thus determined based on respective fold-change when contrasted to controls, to further ensure comparability between analyses performed at different time-points or conditions. Significant gene-trait associations in PheWAS were Bonferroni-corrected. All other statistical analyses were performed using Prism (v9.4.1, GraphPad Software, San Diego, CA, USA). Data were analysed using one- or two-way ANOVA with repeated measures as necessary, followed by software-recommended *post-hoc* tests (Tuckey for one-way, Bonferroni for two-way ANOVA). Normality (D’Agostino-Pearson omnibus) and homogeneity of variances (Brown-Forsythe) were probed and if assumptions were violated, the appropriate tests were performed (Kruskal-Wallis with Dunn’s multiple comparisons test and Welch’s ANOVA with Dunnett’s *post-hoc*, respectively). Statistical significance was accepted for p/q<0.05 and trends were denoted at p<0.08 for behavioural data. All values are presented as mean ± standard error of the mean (S.E.M.).

## RESULTS

### Adult MIA mice display deficits in NPD-related behavioural paradigms

To confirm the validity of our prenatal immune activation model, we generated mouse offspring of both sexes and extensively tested for NPD-relevant behaviours (Figure 1, Supplementary Figure 1–2). First, we established an early-gestation MIA experimental procedure by adapting previously published methods63, utilising the viral mimetic poly(I:C) in two concentrations (*low*, 2.5 mg/kg and *high*, 5 mg/kg; *control*, 0 mg/kg vehicle). The dose-dependent maternal immune response was verified by measuring plasma levels of the pro-inflammatory cytokine Interleukin 6 (IL-6) one hour post injection (Supplementary Figure 1E).

**Figure 2:**
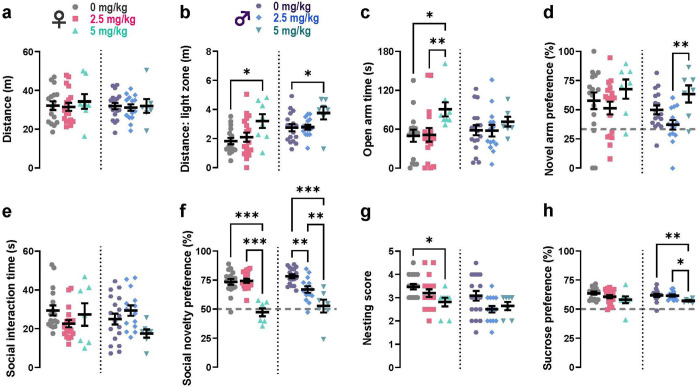
Early maternal immune activation (MIA) distinctly alters relevant behavioural endophenotypes in adult male and female mice. **A,** No differences in locomotion were observed in an Open Field test (one-way ANOVA). **B,** Males and females of the 5 mg/kg MIA-exposed group displayed hyperactivity in the brightly lit compartment of the Light-Dark Box (one-way ANOVA, F: p=0.03, M: p=0.04, Tukey *post-hoc*). **C,** A stronger prenatal immune challenged (5 mg/kg) increased the time spent in the open arm, a measure of reduced anxiety-like behaviour, in an Elevated Zero Maze for females only (Kruskal-Wallis, p=0.008, Dunn’s *post-hoc*). **D,** Different dosage of the prenatal poly(I:C) treatment have divergent impact on the spatial novelty response exclusively in males in a Y-maze test (one-way ANOVA, p=0.004, Tukey *post-hoc*). **E,** When presented with a novel and familiar conspecific of the same sex in a social test, animals of all groups did not spent significantly less time socially interacting (one-way ANOVA, M: p=0.06, Tukey *post-hoc:* trend in males of 2.5 *vs* 5 mg/kg only). **F,** Pronounced deficits in social preference toward the novel mouse were present in offspring of the 5 mg/kg MIA group for both sexes, while only the males of the 2.5 mg/kg group displayed social memory deficits (one-way ANOVA, F: p<0.001, M: p<0.001, Tukey *post-hoc*). **G,** Female offspring of 5 mg/kg poly(I:C)-challenged dams displayed a significant decrease of nest building quality in a two-day nesting test (one-way ANOVA, p=0.04, Tukey *post-hoc*), indicative of self-neglect. **H,** Only males of the 5 mg/kg poly(I:C)- treated group exhibited an anhedonia-like decrease in sucrose preference (Kruskal-Wallis, p=0.01, Dunn’s *post-hoc*). Grey dashed lines were used to denote chance levels of in choice tests. Tested offspring numbers by poly(I:C) treatment dose: *0 mg/kg*, n=16 F, 18 M; *2.5 mg/kg*, n=18 F, 15 M; *5 mg/kg*: n=8 F, 7 M. Significance of the respective *post-hoc* tests denoted as follows: *p<0.05, ^**^p<0.01, ^***^p<0.001, #p<0.08. All data presented as mean ± SEM.

High-dose MIA offspring of both sexes exhibited reduced anxiety-like behaviours, increased distance travelled and time spent in open, brightly lit environments (Figure 2B–D), while retaining normal locomotor function (Figure 2A). The anxiolytic effect of the prenatal high-dose poly(I:C) treatment was more pronounced in females than males in multiple tests (Figure 2B–C, Supplementary Figure S3B-C). No differences were observed in the open-field test, which was used to assess general locomotor activity (Supplementary Figure S3A).

Additionally, the low and high viral mimetic dosages led to divergent spatial novelty preference outcomes, increasing preference in the high- versus low-dose MIA male offspring (Figure 2D). Interestingly, the high-dose treatment group lead to social novelty preference deficits (Figure 2F) in both sexes, in the absence of significant social interaction differences (Figure 2E). Furthermore, depressive-like behaviours in high-dose offspring manifested as self-neglect in females, as assessed in the nest building test (Figure 2G), and anhedonia in males (Figure 2H), assessed in the sucrose preference test. No significant behavioural deficits were observed in the FST (Supplementary Figure S3E).

The ASR was differentially influenced by poly(I:C) dosage in females, so that higher dose MIA-exposed offspring showed an increased startle response (Supplementary Figure S3D). Simultaneously, subtle deficits in sensorimotor gating as measured by PPI of the ASR, were observed in female high-dose MIA offspring (Supplementary Figure S3F). The low-dose poly(I:C) treatment showed moderate effects on some behavioural deficits in males but not females, including significantly reduced social novelty preference (Figure 2F), increased centre time in the open field, and hypoactivity in a novel environment in the Y-maze (Supplementary Figure S3A and S3G).

Taken together, these findings substantiate the model in our laboratory and further support its relevance for NPD-associated behaviours, while revealing sex- and dose-specific effects.

### Sex-specific alterations of the synaptic proteome in adult MIA offspring

**Figure 3:**
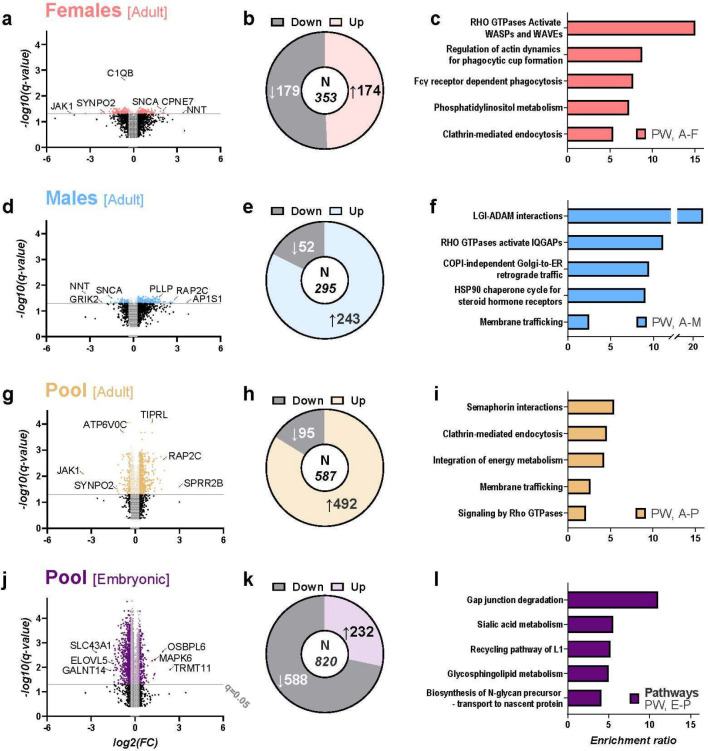
Dynamic alterations of the embryonic and adult synaptic proteome follow prenatal immune activation. Volcano plots of proteomic results from synaptoneurosomes (SN) of early-gestation immune challenged mice (viral-like mimetic poly(I:C), 5 mg/kg) and controls: **A**, adult females (AF); **D**, adult males (AM); **G,** adult pool (AP); **J,** embryonic pool (EP, E18; adults: n=2 M, 2 F each control and treated, 4 AP replicates per group; embryos: n=16 M, 14 F each control and treated animals; 4 EP replicates per group). These show all detected proteins, based on the significance (q-value) and fold-change (FC) in hippocampi of maternal immune activation (MIA) offspring when compared to controls. Selected proteins shown by name in the volcano plots exhibit a combination of high significance, FC, and biological relevance. Colour denotes significant changes (q<0.05), shaded boxes indicate small change in regulation (absolute FC below 0.2). Of those meaningfully altered SN proteins, upregulation was observed in adults for nearly half in females (**B**) and most in males and pool (**E, H**), while the majority of changes in the embryonic pool were downregulations (**K**). The functional involvement of prenatal immune challenge-influenced SN proteins in molecular pathways was investigated further for all groups (*right column*). The pathway (PW) enrichment ratios for MIA-altered proteins of adult females (**C**) and especially males (**F**) were highest, the results indicating sex-specific activation of parallel but distinct cellular response to stimuli and synaptic organisation. Enriched terms for the pooled adult samples largely mirrored the pathway categories from the male and female-specific analyses, including membrane trafficking, trans- and intracellular signalling, as well as neurite guidance (**I**). The dynamic nature of the SN proteome alterations following early prenatal immune challenge is clearly visible from the embryonic data, including the lack of significant sex effects before birth. The altered proteins were involved in stress responses, neuronal development, post-translational modification of membrane proteins, and lipid metabolism (**L**). Significance threshold for Benjamini-Hochberg multiple-test corrected *p*-value) was set at q=0.05 (*t*-test, *volcano plots*; Fisher’s exact test, *pathways*).

To interrogate the functional molecular consequences of prenatal inflammation in the HPC, we investigated the SN HPC proteome using mass spectrometry following the high dose as it resulted in a larger behavioural phenotype. The functional relevance of changes, when compared to control mice, was examined by enrichment analyses in the protein-coding genome and at the synapse, specifically. Of the 3 491 detected proteins, 587 were significantly altered (q<0.05, |change|>0.2), the large majority (83.8 %) of which were upregulated in the adult MIA-exposed offspring (Figure 3G–H, Supplementary Table S3). Functionally enriched pathways of the protein-coding genome included those involved in axon guidance, vesicle-mediated membrane transport, intracellular metabolic regulation, and signal transduction (Figure 3I, Supplementary Figure S5A). Closer inspection of synaptic mechanisms affected by MIA highlighted organisation and structure, protein translation and receptor regulation specifically at the post-synapse (Supplementary Figure S4D, S4G).

Considering the role of sex in animal behaviour, as well as prevalence differences of NPDs and comorbid psychiatric conditions, we wanted to further examine possible sex-dependent effects of MIA on the synaptic proteome. Indeed, 47.8 % of significantly up- or downregulated proteins in the adult pool were sex-specific (Figure 3A, 3D, Supplementary Figure S4A, Supplementary Table S3–4). Moreover, some of the proteins changed in both male and female MIA offspring (e.g., NNT, SNCA) were regulated in opposite directions, while other protein abundance differences were exclusive to either females or males (n=195 and 171, respectively; Figure 3A, 3D, Supplementary Figure S4A, Supplementary Table S3). In females, 50.7 % of the 353 significantly altered SN proteins were downregulated, while in males a large majority (n=243/295) were upregulated (Figure 3B, 3E, Supplementary Table S3–4). Overrepresented molecular pathways in the females included actin cytoskeleton regulation related to the innate immune system and lipid metabolism (Figure 3C, Supplementary Figure S5B). In males, enriched pathways were involved in synapse formation and maturation, hormone-mediated stress responses, and myelin sheath aberrations (Figure 3F, Supplementary Figure S5C). Importantly, in males and females MIA seems to affect parallel but distinct cellular pathways responsible for membrane trafficking, as well as regulation of synaptic cytoskeletal re-organisation by different downstream effectors of Rho GTPases (Figure 3C, 3F). In summary, the observed differences in the HPC synaptic proteome of males and females might explain the sex specificity of behavioural phenotypes.

### MIA alters the prenatal synaptic proteome in a sex-independent manner

Next, we wanted to explore the role of MIA on SN neurodevelopment. Thus, the late embryonic (E18) HPC SN proteome of high-dose MIA male and female offspring was probed for protein changes. Here, 820 proteins were significantly altered by MIA, most (71.7%) of which were downregulated (see Figure 3J–K, Supplementary Table S5). Synapse-level enrichment encompassed pre- and postsynaptic structural organisation, modulation of chemical transmission, and the synaptic vesicle cycle (Supplementary Figure S4E, S4H). Affected protein-coding genome pathways include cellular responses to environmental stress and protein metabolism, such as different post-translational protein modifications related to developmental and membrane function, and sialic acid metabolism (Figure 3L, Supplementary Figure S5D). MIA appears to prominently affect the embryonic lipid metabolism at the synapse, i.e., the synthesis of glycosphingolipids, essential for nervous system development and functionality.

Notably, no MIA-induced sex-specific differences in protein abundance crossed the significance threshold in the embryonic tissue when compared to controls, indicating that divergent effects on the male and female synaptic proteome occur later in life. Here, we investigated poly(I:C) dose-dependent effects on prenatal synaptic neurodevelopment. Indeed, 375 proteins were significantly influenced in the lower-dose MIA offspring when compared to controls, of which 83.2% were downregulated (Supplementary Figure S4C). Here, significantly affected pathways centred around the RNA metabolism, specifically splicing and post-transcriptional cytoplasmic transport, indicating more transient synaptic responses to the low dose immune challenge (Supplementary Figure S4F, S5E). The majority of the observed changes (61.1 %) comprised proteins different from those altered by a high-dose treatment when compared to controls (Supplementary Figure S4B, Supplementary Table S6). Indeed, when contrasting the SN proteome composition between the two poly(I:C) treatments directly, 1 035 proteins differed significantly, of which 59.6 % were upregulated in the high-dose MIA-exposed offspring (Supplementary Figure S4B, S4I). Functional differences involve cytoskeleton remodelling, neuronal development, and differentiation (Supplementary Figure S5F). 444 of those proteins differentially altered by the high and low poly(I:C) treatment were not affected for either prenatally challenged group compared to controls, which might underlie some of the observable phenotypic divergences in low-versus high-dose MIA offspring.

Collectively, our results show significant modifications of the embryonic synaptic proteome during a relevant developmental time-window in late gestation after MIA, which are dose- but not sex-dependent.

### Age-independent synaptoneurosome changes associate with NPD-related phenotypes and environmental stimuli

**Figure 4:**
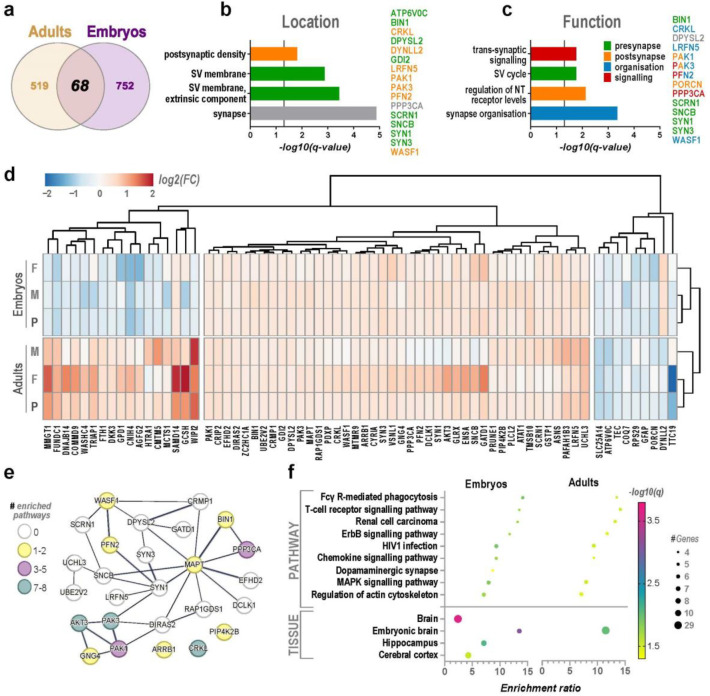
Persistent maternal immune activation (MIA)-induced protein changes at the synapse involve environmental signal and inflammatory responses. Overlap of proteins altered in synaptoneurosomes of both embryonic and adult hippocampi of MIA offspring (**A**), reveal the long-term consequences of a viral-like immune system stimulation during early gestation. The 68 overlapping proteins were enriched at the synapse (**B**), specifically in the postsynaptic density and the synaptic vesicle membrane (SV). Functionally, these proteins are significantly involved in the SV cycle, neurotransmitter (NT) receptor and signalling regulation, and synaptic organisation (**C**). Colours for the overrepresented proteins shown in **B-C** match those of the respective enriched term category (legend in C, *multiple colours=synaptic protein included in multiple functional terms*). Hierarchical clustering of the respective average fold-change (FC) for each time-point for the 68 long lastingly MIA-affected proteins revealed similarities for three protein groups, namely downregulated in adults, mostly downregulated in embryos and upregulated in adults, and persistently upregulated in both (**D**: F, female; M, male; P, pool, adults: n=2 M, 2 F each control and treated, 4 P replicates per group; embryos: n=16 M, 14 F each control and treated animals; 4 P replicates per group). The latter of those, showing long-term upregulation, formed a highly significant interaction network (p=9.82 × 10–07) of proteins (**E**). A number of those proteins, like AKT3, CRKL, PAK1, PAK3, PPP3CA, and others, were part of multiple functionally enriched pathways. Pathway enrichment in both embryos and adults underscored the involvement of this subnetwork’s embryonic brain-enriched common proteins in the cellular response to the environmental stimuli and inflammatory stress via different signalling cascades, and cytoskeletal organisation (**F**). Additionally, proteins from this cluster network are enriched in the hippocampus and glutamatergic synapse, crossing significance threshold in the embryonic dataset. Significance was set at q<0.05 for all tests (Benjamini- Hochberg multiple-test corrected *p*-value).

Next, we wanted to explore the dynamic influence of early gestational MIA on the developing and mature synaptic proteome. While most of the identified proteomic changes in the HPC following high-dose MIA exposure were age-specific, 68 alterations of proteins discovered in the embryos were common to the adult pool (Figure 4A). Synapse-level analyses revealed these proteins to be enriched at the postsynaptic density and synaptic vesicle membrane, and their functional role in synapse organisation and signalling (Figure 4B–C).

To further investigate these common proteins, we performed a hierarchical cluster analysis (Figure 4D), which confirmed the similarity among the embryonic samples but a clear separation from those observed in the adult HPC. In adult MIA offspring, the pool appears to be most strongly driven by protein changes in the females, while the embryonic pool resembles more closely the male proteomic profile (Figure 4D). The interrogated proteins formed three major clusters, namely mostly down- or upregulated at both ages, and those overwhelmingly decreased in embryos but increased in adults. Most probed proteins were upregulated at both time-points.

Subsequently, we interrogated protein-protein-interactions for the common protein dataset in a publicly available bioinformatics database (STRING). The analysis revealed a network of significantly interconnected proteins in the second, persistently upregulated cluster of 42 proteins (interaction enrichment p=7.65e-07, Figure 4D–E). Within this interaction network, a subset of proteins was highly represented in the identified functional pathways (Figure 4E–F). These included AKT3, CRKL, PAK1/3, and PPP3CA, which are known to be strongly involved in several cellular responses to environmental and pathogenic or inflammatory cues^77–79^. Functional enrichment in the embryonic dataset further highlighted the dopaminergic synapse, while tissue enrichment results confirmed the embryonic brain and the HPC, an element of the cerebral cortex, as structures significantly converging with this persistently upregulated protein cluster (Figure 4E–F).

Finally, we aimed to investigate the extent of association between the revealed MIA-induced, long-term synaptic changes with NPDs and their comorbidities. Therefore, we analysed PheWAS data for the genes encoding the 68 proteins that overlapped between the embryonic and adult proteomes. We determined that all but one of these proteins (porcupine, PORCN) were significantly associated with psychiatric or cognitive traits (Supplementary Table S8–9). The highest correlation significance was revealed for *MAPT*, *PLCL2*, *LRFN5*, *AKT3*, and *PPP3CA*. Apart from NPDs in a narrower sense, i.e. ADHD, ASD, SCZ, and bipolar disorder (BPD), the MIA-linked genes were associated with known comorbid conditions like conduct disorder (CD), obsessive-compulsive disorder (OCD), post-traumatic stress disorder (PTSD), substance use disorders (SUDs), anxiety- and depression-related traits (Table 1, Supplementary Table S8). When considering NPDs of interest, depression, and anxiety alone, the strongest pleiotropic effects of single genes, i.e. highest number of associations with disorders, were observed for *PFN2*, *HTRA1*, *SCRN1*, *ATAT1*, and *SYN3* (Supplementary Figure S6). Memory, higher cognitive functions, and mild intellectual disability were similarly among the phenotypic categories significantly associated with many of the probed genes (Table 1, Supplementary Table S8–9). Other significant gene-trait relationships encompassed temperament and personality traits like neuroticism, risk taking, aggression, irritability, mood swings, as well as sleep, attention, and psychomotor functions.

Taken together, our results indicate that a modest subset of synapse- and signalling pathway-enriched proteins, which are changed prenatally and in the adult HPC following MIA, might play a causal role in the increased risk of emergence of NPDs and related nervous system sequelae after a prenatal viral infection.

## DISCUSSION

In the current study, we examined and directly compared dynamic and persistent proteomic changes in embryonic and adult gestationally immune-challenged offspring of both sexes. Here, we demonstrate sex-specific effects in adult, but not embryonic, SN HPC proteome of MIA-exposed mice. Importantly, a subset of 68 proteins which were consistently and age-independently affected by MIA in mature and prenatal HPC SN were associated with NPD-related phenotypes, and environmental stimuli. These proteins may represent targets for tailored approaches for early treatment in individuals at high-risk or after early diagnosis.

Maternal immune system activation during gestation increases the risk for developmental perturbations leading to subsequent neuropsychiatric disorders in offspring^80^. The optimal doses of the viral mimetic poly(I:C) have to be determined for each laboratory environment and we initially demonstrated that while 2.5 and 5 mg/kg were viable in our facility, higher doses that are also widely used, such as 7.5 NS 10 mg/kg led to litter losses ^81,82^. While both viable doses led to NPD-relevant endophenotypes, such as social deficits, the higher (5 mg/kg) dose caused more robust behavioural manifestations, the lower dose led to less discernible aberrations and only in male offspring. Taken together, these findings suggests that male offspring are more susceptible to the MIA paradigm, with the behavioural consequences only observed in both sexes at the higher dose. Therefore, this dose was selected to compare the behavioural findings with alterations in the HPC SN proteome.

When selecting a brain region for molecular investigations, we considered the observed MIA endophenotypes and the role of the HPC in learning and memory, NPD-relevant social behaviors^19,83,84^ and novelty response^21^, and its life-long plasticity. Few studies have examined HPC changes after immune activation in early gestation^44,55^, although the region has been mechanistically implicated in dysfunction following MIA^22,24,47^. Moreover, we were unable to find data-driven investigations of the enriched synaptic proteome of MIA offspring, despite the emerging central role of the synapse in neuroinflammation and psychiatric illness^10,14,29,32,33,46,51,85,86^.

Our findings of extensive prenatal synaptic proteome modifications in offspring of 5 mg/kg poly(I:C)-treated dams implicate lipid and protein metabolism pathway disruptions during a relevant neurodevelopmental time-window for possible lasting NPD-pertinent sequelae^87^. Tissue extraction in a large subset of offspring shortly before birth (i.e. E18) allowed for an examination at a time after supportive cells involved in the immune and cellular responses, i.e. astrocytes and oligodendrocytes, have differentiated61 but before the perinatal excitatory-to-inhibitory gamma-aminobutyric acid (GABA) switch, which is affected by MIA^88^, is completed. Gestational neuroinflammation affected the neuromembrane before birth, including degradation of cell junction proteins relevant for neuronal guidance^89^ and impairs glycosphingolipid metabolism important for cell signalling and myelination^90^. In the embryos, we revealed MIA-induced changes in proteins involved in post-translational modifications by glycans and sialic acid, which affect membrane bio-properties and proteins controlling the secretory pathway, intercellular interactions, and developmental timing^91–93^. Interestingly, a study of the transcriptome of stimulated microglia in an adult early-gestation MIA dataset found enrichment of fatty acid metabolic and protein localisation pathways in poly(I:C)-treated offspring46. Direct comparison of our data with previous findings in the embryonic brain remain difficult due to the differences in experimental design, notably MIA and foetal brain extraction timing, compound type and concentration, the samples’ brain region, sex, and cellular composition. Nevertheless, published results in embryonic MIA brains describe pathway enrichment for cellular and immune responses, transmembrane signalling, and axon guidance^36,38^, networks of neuronal development and metabolic or energy processes^39^, and cytoplasmatic translation^48^ in keeping with the present findings.

In adulthood, the MIA-induced dynamic changes in mature HPC SN had largely shifted to divergent transmembrane trafficking processes, intracellular signalling cascades, and hormone-mediated energy metabolism (see Figure 3, Supplementary Figure S5 for full details). Our data-driven protein analysis combined male and female adult mice, while most large-scale transcriptomic studies were restricted to males^16,41–43,47,50^. Published findings studying HPC from both sexes revealed disruptions in parallel, converging growth factor signalling pathways in adolescent^44^ and adult^55^ mice following an early-gestation poly(I:C) challenge. Growth factor receptors are coupled to receptor tyrosine kinases, which act on Rho GTPases, enriched in our dataset, affecting downstream intracellular signalling cascades and revealing a robust MIA effect on signal transduction across the postnatal period. Since multi-omics have revealed differences in the ventral and dorsal HPC in both stress and MIA models^44,56^, the exact site of the HPC analysis might need to be considered in future detailed studies of MIA effects on the proteome.

Detection of a larger number of proteins for the embryonic dataset (n=6 435 *vs* 3 511 for adults) corresponds to higher amounts of tissue in pooled samples as well as of analysed peptides, the immature state of the synapse influencing the SN composition and isolation, or marginally higher sensitivity of mass spectrometry equipment used. To explore the comparability of data further, we randomly selected five proteins only detected in adults (5.1% of detected protein set), for which or whose paralogs data was available in the Allen Brain Atlas for the developing mouse brain^94^. We compared the expression profiles between the late embryonic (E15.5–18.5) and postnatal (P14–28) brain. For the five probed proteins (GRIN2A, KCNJ6, LRRTM1/2, MOBP, SYT1/10) corresponding gene expression in the telencephalic vesicle, giving rise to the HPC, was significantly higher after birth. This suggests that the differences in the numbers of identified proteins might be rooted in physiological, dynamic changes in expression during brain tissue development and maturation.

The proteomic alterations observed in the present study are significantly associated with psychiatric disorders and contain a cluster of upregulated proteins that build an enriched functional network. Persistently changed interacting proteins are involved the cellular and immune responses to environmental stimuli38 and glutamatergic synapse signalling, in line with previous findings. Indeed, effects of a prenatal immune challenge have been consistently observed in glutamatergic neurons ^29,35,42,47,55^, even across multiple generations^43^. Furthermore, previous studies across different ages, brain regions, and MIA model designs have highlighted different molecules along the converging signalling cascades in cellular kinase pathways^16,42–44^ such as the ERK and mTOR pathways that are heavily implicated in NPDs and treatment responses^95–97^. Taken together, the persistent nature of synaptic changes, enrichment in the embryonic brain, and the importance of affected signalling pathways for brain development and NPDs^97,98^, motivated us to probe their association with psychiatric and cognitive traits. While previous transcriptomic studies of the MIA model have repeatedly linked immune challenge-caused gene dysregulation to NPDs^8,10,16,46,51^, the degree of phenotypic association and number of proteins with pleiotropic effects in our dataset was compelling. It should be noted that enrichment of synaptic processes and compartments, as well as associations with psychiatric and cognitive traits in the investigated datasets is to be expected, considering the implementation of SN isolates. Nevertheless, our findings reveal MIA effects on important molecular pathways and repeatedly altered, highly NPD-associated synaptic proteins, considering that all results directly compare treated offspring to controls using the same conditions. Given the enduring changes in such proteins, and their relevance for NPDs, we suggest that these proteins, and related pathways, represent novel early-intervention targets. While the embryo-specific changes might occur too early and adult-specific changes might be too late for intervention, these persistently changed proteins might be targeted throughout the developmental trajectory.

Moreover, our study suggests that significant sex-specific, synaptic-level effects following MIA emerge later in life, possibly following known influences such as chromatin remodelling, environmental, and hormonal regulation^99,100^. Previous findings from the limited number of studies on sex influencing embryonic transcriptome changes following MIA are inconclusive or non-comparable with our data due to experimental differences^39,48,54,55^. Sex-related differences observed in other embryonic studies using whole-brain or full tissue samples may be underlain by MIA effects on proteins that are enriched outside of the synapse or in immune brain cells^101^, and RNA alterations potentially fail to translate into functional protein changes^102^. Our findings imply that adult males and females differ in their structural synaptic response to the early prenatal immune challenge. This hypothesis is in line with the sex-dependent innate immune response^103^ and whole-cell transcriptomic results from previous studies in HPC of male and female offspring from a comparable MIA model^44,55^. Protein alterations exclusive to males are involved in steroid-hormone stress responses^103,104^ and in myelination, in concurrence with previous experiments ^41,52,58^. The pathways selectively changed in the rarely investigated females pertain to immune system-related phagocytosis, actin binding, and filament organisation, possibly linked to oestrogen effects^105^. Interestingly, significantly enriched Rho-GTPase signalling pathways were found in both sexes but activate different downstream effectors causing distinct cytoskeletal remodelling, namely WASPs/WAVEs^106^ in females and IQGAPs^107^ in males. Since Rho-GTPases are major dendritic and synaptic regulators^108^, sex-specific functional differences in their target activation might explain divergence in observed NPD- and comorbidity-linked behaviours. Indeed, psychiatrically relevant sex differences in stress responses via divergent neuronal GTPase signalling pathways have been proposed as a concept for sex-dependent vulnerabilities.^109^.

Additionally, we discovered novel dose-dependent alterations in foetal synaptic protein composition with lower viral mimetic dosage [2.5 mg/kg poly(I:C)] influencing alternative splicing and RNA metabolism, which constitute more transient neuronal changes. The proteomic differences between the high and low dose embryonic groups were pronounced, indicating divergent pathways involved in MIA-evoked immune response based on severity. Adult males might be more susceptible than females to NPD-like behavioural symptoms following low-dose MIA as a consequence of female-specific compensatory mechanisms^99^ in the developing brain, which can rectify the effects a mild but not severe immune challenge. Akin to the investigation of embryonic and adult HPC transcriptome changes following chronic MIA exposure by Nakamura *et al.*^55^, future research might assess the consequences of a single-, low-dose immune challenge on the HPC proteome in both sexes.

In conclusion, this investigation of the HPC synaptic proteome provides insights into the dynamic effects of an early prenatal immune challenge on the developing and mature brain. The observed dynamic alterations at the synapse were age-specific and varied by sex or immunostimulant dose in the adult and embryonic brain, respectively. Simultaneously, we discovered a smaller but highly relevant subset of embryonic SN changes caused by MIA that persist into adulthood, which could represent targeted approaches in high-risk or early diagnosis individuals. The provided evidence infers a causal link between altered synaptic proteins, neurodevelopmental pathways, and related psychiatric endophenotypes.

## Figures and Tables

**Table 1.** Differentially expressed genes, encoding proteins changed before birth and in adult prenatally immune challenged offspring, are strongly correlated with psychiatric conditions and mental functions. A publicly available phenome-wide association study (PheWAS) database was used to interrogate the causal links of persistently changed genes and NPDs and comorbidities of interest. Full lists of significantly associated disorders and traits for each gene, including statistics and sample sizes, can be found in Supplementary Tables 8–9.

Condition or Function	Significantly correlated genes PheWAS analysis summary
**ADHD**	*ATAT1, BIN1, COQ7, CRMP1, CYRIA, DCLK1, FTH1, GATD1, GLRX, HTRA1, LRFN5, MAPT, MTMR9, PFN2, PLCL2, RAP1GDS1, SNCB, SYN3, VSNL1, WASHC4*
**CD**	*FTH1, LRFN5, PIP4K2B, PPP3CA, PRUNE1, RAP1GDS1*
Anxiety* **| Neuroticism**	*AGFG2, AKT3, ASNS, ATAT1, BIN1, CMTM5, CNIH4, COMMD9, COQ7, CRIP2. CRMP1, CYRIA, DCLK1, DKK3, DPYSL2, DYNLL2, EFHD2, FTH1, GDI2, GFAP, GNG4, GPD1, GSTP1, HTRA1, LRFN5, MAPT, MTMR9, PAFAH1B3, PIP4K2B, PLCL2, PPP3CA, PRUNE1, RAP1GDS1, SCRN1, SNCB, SYN3, TEC, TMSB10, TRIAP1, TTC19, UCHL3, VSNL1, WASF1, WIPI2, ZC2HC1A*
**ASD**	*CRKL, CRMP1, DCLK1, GLRX, GPD1, HTRA1, MAPT, MTMR9, PDXP, PFN2, PPP3CA, SCRN1, TRIAP1, WIPI2*
**BPD**	*AKT3, ATAT1, CNIH4, CRKL, CYRIA, DIRAS2, DNAJB14, ENSA, GDI2, GFAP, HTRA1, PAFAH1B3, PAK1, PFN2, PRUNE1, RAP1GDS1, RPS29, SAMD14, SCRN1, SYN3, TMSB10, WASF1, WASHC4*
**Depressive Disorders**	*AGFG2, AKT3, ARRB1, ASNS, ATAT1, ATP6V0C, BIN1, CMTM5, COMMD9, COQ7, CRIP2, CRKL, CRMP1, CYRIA, DCLK1, DIRAS2, DKK3, DNAJB14, DPYSL2, DYNLL2, EFHD2, ENSA, FTH1, GATD1, GCSH, GDI2, GFAP, GLRX, GNG4, GPD1, GSTP1, HTRA1, LRFN5, MAPT, MMGT1, MTMR9, PAFAH1B3, PAK1, PFN2, PIP4K2B, PLCL2, PPP3CA, PRUNE1, RAP1GDS1, RPS29, SAMD14, SCRN1, SLC25A14, SNCB, SYN3, TEC, TRIAP1, UBE2V2, UCHL3, VSNL1, WASF1, WASHC4, WIPI2, ZC2HC1A*
**Memory | Cognition** **	*AGFG2, AKT3, ARRB1, ASNS, ATAT1, BIN1, CMTM5, CNIH4, COQ7, CRIP2, CRMP1, CYRIA, DCLK1, DKK3, DPYSL2, DYNLL2, EFHD2, ENSA, FTH1, FUNDC1, GCSH, GDI2, GFAP, GLRX, GNG4, GPD1, HTRA1, LRFN5, MAPT, MCTS1, MTMR9, PAK1, PAK3, PDXP, PFN2, PIP4K2B, PLCL2, PPP3CA, PRUNE1, RAP1GDS1, RPS29, SAMD14, SCRN1, SNCB, SYN3, TMSB10, TRIAP1, TTC19, UBE2V2, UCHL3, VSNL1, WASF1, WASHC4, WIPI2, ZC2HC1A*
**OCD**	*AKT3, DPYSL2*
**PTSD**	*AGFG2, AKT3, ARRB1, COMMD9, CRKL, DKK3, MAPT*
**SCZ | SCZ vs BPD**	*AGFG2, AKT3*^&^, *ASNS*^&^, *ATAT1*^&^, *ATP6V0C, BIN1, CNIH4, COMMD9, COQ7*^$^, *DIRAS2*^$^, *DCLK1*#^&^, *DKK3, DNAJB14*^&^, *DPYSL2, EFHD2*^#^, *ENSA*^#^, *FUNDC1*^#^, *GNG4, GPD1, LRFN5*^#^,^&^, *MAPT, MTMR9, PAFAH1B3, PDXP*^#^, *PFN2*^$^, *PIP4K2B, PLCL2, PPP3CA, PRUNE1*^&^, *RPS29, SAMD14*^$^, *SCRN1, SYN1*^#^, *SYN3*^&^, *TEC*^#^, *VSNL1*^&^, *WASHC4*^#^

* Anxiety includes traits containing “Anxiety” and “Anxious”.

** Memory comprises “Memory Functions” and “Dementia in Alzheimer’s Disease” subchapters, Cognition corresponds to “Higher-Level Cognitive Functions” and “Mild Mental Retardation” *(updated to Mild Intellectual Disability in text).*

# SCZ only,

$ SCZ vs BPD only,

& incl. Psychiatric Genomics Consortium (PGC) cross-disorder. *ADHD,* Attention Deficit Hyperactivity Disorder; *CD,* conduct disorder; *ASD,* Autism Spectrum Disorder; *BPD,* Bipolar Disorder; *OCD,* Obsessive Compulsive Disorder; *NPD,* Neurodevelopmental Psychiatric Disorder; *PTSD,* Posttraumatic Stress Disorder; SCZ, Schizophrenia

## Data Availability

The mass spectrometry proteomics data have been deposited to the ProteomeXChange Consortium via the PRIDE partner repository (PMID: 34723319) with the dataset identifier PXD043094. Synaptic proteome analysis files are available on Figshare (licence CC-BY 4.0) with the following DataCite DOIs, pending acceptance: https://doi.org/10.6084/m9.figshare.22581628 for embryonic dataset analyses, https://doi.org/10.6084/m9.figshare.22581766 for adult dataset analyses. All other data supporting this study’s findings is presented either in the main text or supplementary tables, figures, and files.
